# The impact of the MIB-1 index on facial nerve outcomes in vestibular schwannoma surgery

**DOI:** 10.1007/s00701-020-04283-z

**Published:** 2020-03-09

**Authors:** Johannes Wach, Simon Brandecker, Agi Güresir, Patrick Schuss, Hartmut Vatter, Erdem Güresir

**Affiliations:** Department of Neurosurgery, University of Bonn, Sigmund-Freud Straße 25, 53127 Bonn, Germany

**Keywords:** Extent of resection, Facial nerve, House-Brackmann, MIB-1, Vestibular schwannoma

## Abstract

**Background:**

Facial nerve palsy is a severe morbid condition that occurs after vestibular schwannoma (VS) surgery. The objective of this study was to evaluate facial nerve outcomes based on surgical techniques, tumour size, and immunohistochemical factors.

**Methods:**

One hundred eighteen patients with VS were retrospectively analysed. Gross total resection (GTR) was achieved in 83 patients, and subtotal resection (STR) was achieved in 35 patients. Follow-up was 60 months (median). Facial nerve outcomes were assessed for 24 months after surgery. Analysis of the MIB-1 index was performed in 114 patients (97%) to evaluate recurrence and facial nerve outcomes.

**Results:**

Immediately after surgery, 16 of 35 patients (45.7%) with STR and 21 of 83 patients (25.3%) with GTR had a good (House-Brackmann (HB) score ≤ 2) facial nerve outcome (*p* = 0.029). Semi-sitting positioning (*p* = 0.002) and tumour size class of 3 (> 4 cm) were also associated with worse HB outcomes after 2 years (*p* = 0.004) in univariate analyses. The MIB-1 index was significantly correlated with diffuse infiltration of tumour-associated CD45^+^ lymphocytes (*r* = 0.63, *p* = 0.015) and CD68^+^ macrophages (*r* = 0.43, *p* = 0.021). ROC analysis found an AUC of 0.73 (95% CI = 0.60–0.86, *p* = 0.003) for the MIB-1 index in predicting poor facial nerve outcomes. Binary logistic regression analysis revealed an MIB-1 index ≥ 5% (16/28 (57.1%) vs. 5/40 (12.5%); *p* < 0.001, OR = 14.0, 95% CI = 3.2–61.1) and a tumour size class of 3 (6/8 (75.0%) vs. 2/8 (25.0%); *p* = 0.01, OR = 14.56, 95% CI = 1.9–113.4) were predictors of poor HB scores (≥ 3) after 1 year.

**Conclusions:**

An MIB-1 index ≥ 5% seems to predict worse long-term facial nerve outcomes in VS surgery.

## Introduction

Vestibular schwannoma (VS) is a benign tumour that occurs in the cerebellopontine angle, and its origin is the Schwann cell cover of the eighth cranial nerve. VSs are the most common cerebellopontine angle tumours and account for 75% of all lesions in this location [3]. It has been claimed that gross total resection (GTR) is the optimal surgical strategy to achieve good tumour control in VS patients [33].

A few centres assume that it is plausible to perform a planned subtotal or near total resection before irradiating the tumour [23]. The natural history and appropriate follow-up for residual tumour tissue and the facial nerve are controversial, and it is assumed that it is necessary to perform stringent follow-up with MRI of the residual tumour for at least 7–10 years [12].

The retrosigmoid approach is frequently used and seems to be useful with regard to radical resection and facial nerve function-preserving resection in VS [7, 40]. Furthermore, middle fossa [34] or translabyrinthine approaches can also be applied [3, 9, 35].

Postoperative facial nerve palsy can create many serious side effects. Often, further surgical interventions, such as hypoglossal-facial nerve anastomosis [21] or treatment of eye irritations and tear dysfunctions, are necessary to maintain quality of life [2]. In addition to the influence of surgical and technical advances on the postoperative maintenance of facial nerve function, tumour oedema, the Antoni B/A ratio, and proliferation are also viewed as prognostic factors [14]. Furthermore, an immune response and tumour-associated macrophages are also commonly found in VS and are predominantly located in Antoni B areas [44], with their amount correlated with the duration of baseline symptoms [26], tumour volume [16], and the tumour growth rate [17].

Immunohistochemistry is not a good predictor of the clinical features of VS so far. The Molecular Immunology Borstel (MIB-1) index is an excellent tool for detecting nuclear structures that are exclusively present in proliferating cells. The Ki-67 antigen is present in the nuclei of cells in the G1, S, and G2 phases of the cell division cycle as well as during mitosis. Consequently, the detection of this antigen is a feasible method for determining the growth fraction of a given neoplastic cell population [19, 36].

The MIB-1 index has been reported in some studies to be an important indicator of the regrowth of residual tumours. It has been proposed that in a VS, a MIB-1 index > 2% indicates a shorter tumour doubling time [46]. In addition, it has been suggested that the mean MIB-1 or Ki-67 labelling index will be higher in growing tumours than in static tumours, demonstrating that in VS, relative to growth, there are fewer inflammatory cells [27]. However, the clinical impact of the MIB-1 index on facial nerve outcomes is unknown.

Therefore, we analysed a series of 118 VS patients who underwent surgical resection via a retrosigmoid approach with continuous intraoperative facial nerve monitoring.

## Methods and materials

### Study design and general patient characteristics

From April 2001 to October 2017, 118 patients with VS were treated surgically in our department using the retrosigmoid approach and retrospectively analysed. Patients with prior radiotherapy, second surgery, immediate stereotactic radiosurgery after resection, or neurofibromatosis type 2 were excluded. To improve the comparability of facial nerve outcomes, we excluded all patients with predominantly intrameatal VS who underwent surgery via the translabyrinthine approach. The patients’ ages ranged from 17 to 79 years (mean age and standard deviation (SD), 54 ± 14.3 years). There were 67 female (56.8%) and 51 male (43.2%) patients. Fifty percent of the patients were between 45.75 and 54 years old. This project received ethical approval from the Ethics Committee of the University Hospital of Bonn.

### Surgical technique

All patients in the present series underwent tumour resection using the retrosigmoid approach. Patients were settled in a lateral (86/118; 72.9%) or semi-sitting position (32/118; 27.1%). There were no inclusion or exclusion criteria with regard to the decision to use one of these two positionings. The relative experience of the surgeons was homogenously distributed between the two positioning groups. A linear skin incision was made behind the ear. A retrosigmoid craniotomy was performed to expose the connection between the transverse sinus and sigmoid sinus. After a curved incision was made in the dura mater, cerebrospinal fluid (CSF) was slowly aspirated from the cerebellomedullary cistern to expose the tumour. Intraoperative electromyogram (EMG) monitoring was used in all cases. EMG recordings of the orbicularis oculi and oris muscles were used to monitor the facial nerve. To assess the facial nerve response, a bipolar stimulus with an intensity of 1 to 0.05 mA and a duration of 0.1 ms was used.

### Immunohistochemistry

All surgical specimens were evaluated with haematoxylin/eosin staining and processed for immunohistochemical reactions with antibodies directed against S-100 (Dako, Glastrop, Denmark) as well as monoclonal antibodies against Ki-67 (MIB1; Dako, Glastrop, Denmark). The MIB-1 index was determined in randomly selected high-power microscopic fields. The proportions of stained and unstained nuclei in the tumour cells were determined. The MIB-1 index was defined as the percentage of Ki-67-positive nuclei. Lymphocytic infiltration and macrophages were evaluated using the markers CD45 (Dako, Glastrop, Denmark) and CD68 (Dako, Glastrop, Denmark), respectively.

### Follow-up

The mean and median follow-up times were 60 and 45.25 months, respectively. Pre- and postoperative HB scores were available for all 118 patients. The MIB-1 index was available in 114 of 118 patients. Postoperative HB scores were evaluated after 3, 12, and 24 months. HB scores were available after 24 months in 54 of the 118 patients. Recurrence-free survival and follow-up, including MRI surveillance, were analysed in 62 patients. Patients were retrospectively reviewed for clinical status, operative findings, radiological results, clinical outcomes, and immunohistochemical results. The data were analysed with regard to the extent of resection, tumour size, surgical results, surgical positioning, short- and long-term outcomes of facial nerve palsy, and tumour follow-up.

### Definitions and outcome measures

Patients were analysed separately for tumour size. Tumour size was categorised according to the patient’s largest extrameatal tumour diameter in the cerebellopontine angle on post-contrast axial MR images. A tumour size of class 3 is defined as a diameter > 4 cm, class 2 2–4 cm, and class 1 < 2 cm [20]. Tumour size class 2 (79/118, 67%) was more common than tumour size classes 1 (20/118, 17%) and 3 (19/118, 16.1%). GTR was defined as complete resection without any residual nodular enhancement. Subtotal resection (STR) was defined as an extent of resection (EoR) of 90–99% of the tumour [18]. The extent of resection was assessed by independent neuroradiologists using pre- and postoperative 1.5 or 3.0 Tesla MR images. GTR was achieved in 70.3% of all cases. The facial nerve outcomes and HB scores were dichotomised. Good was defined as an HB score ≤ 2, and poor was defined as an HB score ≥ 3.

Recurrence was defined as the progression of the tumour with an increase over the previous tumour volume of > 20% or a size 2 mm larger than that found on the previous MRI and the intention to treat the tumour. Therefore, 62 patients were analysed with a median follow-up of 60 months.

Facial nerve palsy-induced morbidity was analysed across the three tumour size classes. Postoperative VP shunt dependency was evaluated in all patients. The MIB-1 index was also analysed in 114 patients with regard to recurrence and facial nerve outcomes after 1 year. The patients were dichotomised into 2 groups according to the MIB-1 index using previously reported cut-off points (MIB-1 index ≥ 5% and < 5%) [1], and the results of a receiver operating characteristic (ROC) analysis were analysed with regard to the prediction of recurrence and facial nerve outcomes.

### Statistical analysis

The number of patients with a good facial nerve outcome was examined across different groups separated according to the extent of resection, tumour size, surgical positioning, and the MIB-1 index. VP shunt dependency was statistically analysed in all tumour size classes and resection groups. Pearson’s chi-squared tests (two-sided) and Fisher’s exact tests (two-sided) were used to detect significant differences. The Kaplan-Meier analysis was performed to compare the two groups according to the extent of resection. Differences were analysed using the log-rank test. Two-sample *t* tests were used to compare mean values. ROC curves were calculated to determine the area under the curve, specificity, and sensitivity of the MIB-1 index for predicting facial nerve outcomes according to House-Brackmann scores. Univariate and binary logistic regression analyses were performed to determine the impact of the MIB-1 index on facial nerve outcomes. Statistical significance was assumed when *p* was < 0.05.

## Results

### Extent of resection and outcomes

Immediately after surgery, the HB score was good in 45.7% of patients in the STR group vs. 25.3% in the GTR group (*p* = 0.029). With regard to postoperative useful hearing, no difference was observed (*p* = 0.50). Recurrence rates did not differ between the STR (2/23) and GTR (3/39) groups (log-rank test: *p* = 0.69). Clinical outcome data are shown in Table 1.

**Table 1:** Impact of the extent of resection on facial nerve outcomes, auditory nerve function, and recurrence

	GTR	STR	*p* values
HB good (≤ 2) preoperatively	80/83 (96.4%)	30/35 (85.7%)	0.035
HB good (≤ 2) postoperatively	21/83 (25.3%)	16/35 (45.7%)	0.029
HB good (≤2) at 3 months	25/45 (55.6%)	19/28 (67.9%)	0.296
HB good (≤ 2) at 12 months	29/38 (76.3%)	20/27 (74.1%)	0.836
HB good (≤ 2) at 24 months	29/34 (85.3%)	16/20 (80.0%)	0.614
Useful hearing preoperatively	50/83 (60.2%)	18/35 (51.4%)	
Useful hearing postoperatively	20/83(24.1%)	10/35 (28.6%)	0.488
Recurrence estimator in months	59.2	58	0.692
95% confidence interval, lower limits of recurrence	57.5	52.7	
95% confidence interval, upper limits of recurrence	60.9	63.3	

### Tumour size classes

Facial nerve outcomes were compared among the tumour size classes. Patients with a large VS (tumour size class 3) had a worse postoperative facial nerve outcome, which was statistically significant immediately postoperative and at the 12- and 24-month follow-up examination. Further results are summarised in Table 2. The rate of GTR was 95% (18/19) in the tumour size in class 1 group and significantly higher compared with the tumour size in classes 2 and 3. Fisher’s exact test (two-sided) revealed that with regard to GTR, there was a significant difference between the tumour size classes 1 and 2 (95% (18/19) vs. 67.1% (53/79), *p* = 0.01). Comparison of rates of GTR between tumour size classes 1 and 3 (95% (18/19) vs. 57.9% (12/20); *p* = 0.006) revealed also a significant difference.

**Table 2:** Tumour size classes: comparison of facial nerve outcomes and rates of gross total resection using Fisher’s exact test (two-sided)

Variable	Tumour sizelass 1 (< 2 cm)	Tumour size class 2 (2–4 cm)	Tumour size class 3 (> 4 cm)	*p* values Tumour size classes 1 and 2 (< 4 cm) vs. tumour size class 3 (> 4 cm)
HB good (≤ 2) preoperatively	18/19 (94.7%)	74/79 (93.7%)	19/20 (95.0%)	0.99
HB good (≤ 2) postoperatively	6/19 (31.6%)	29/79 (36.7%)	2/20 (10.0%)	0.03
HB good (≤2) at 3 months	8/10 (80.0%)	34/55 (61.8%)	2/8 (25.0%)	0.05
HB good (≤ 2) at 12 months	7/9 (77.8%)	38/46 (82.5%)	1/6 (16.7%)	0.01
HB good (≤ 2) at 24 months	7/9 (77.8%)	35/41 (85.4%)	2/6 (33.3%)	0.01

Facial nerve palsy–induced morbidity was more common for tumour size class 3 (15.8% (tumour size class 3) vs. 6.3% (tumour size class 2) vs 0% (tumour size class 1); Fisher’s exact test (two-sided): tumour size class 3 vs. tumour size class 1 (*p* = 0.06) and tumour size class 3 vs. 2 (*p* = 0.18)). The overall rate of facial nerve palsy-induced morbidity was 6.8% (8/118) in all tumour size classes. There were 2 cases of corneal scars, 2 cases of hypoglossal-facial nerve anastomoses, 3 cases of gold implants in the upper eyelid, and 1 case of corneal ulcer.

### MIB-1 index and macrophages-lymphocytes

Immunohistochemical analysis for MIB-1 was available in 114 patients (96.6%). The overall median (range) MIB-1 index was 4.0 (1.0–9.0). The mean MIB-1 index in VSs with and without recurrence was 4.16 (SD: 1.2) and 4.00 (SD: 0.63), respectively (*p* = 0.61).

**Fig. 1:**
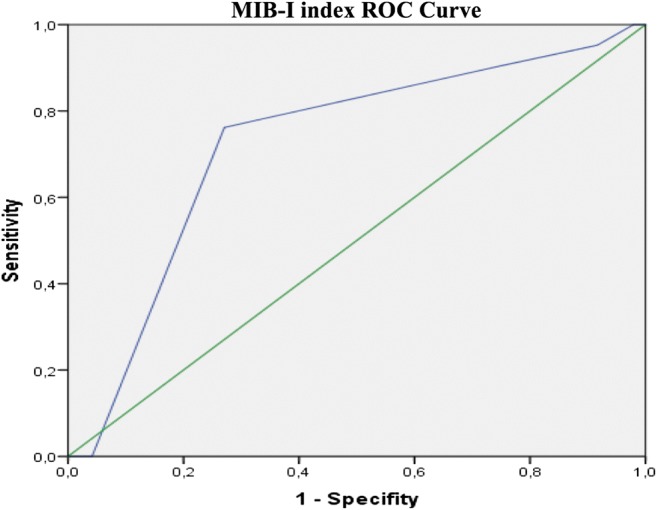
Receiver operating characteristic (ROC) curve illustrating the performance of the MIB-1 index in the prediction of poor facial nerve outcomes (House-Brackmann score > 2). AUC 0.73 (95% CI = 0.60–0.86, *p* = 0.003)

The association between the MIB-1 index and facial nerve outcomes after 12 months was analysed. An ROC analysis of the MIB-1 index was applied to our study patients and revealed an AUC of 0.73 (95% CI 0.60–0.86; SE 0.068, *p* = 0.003), indicating good discrimination regarding good and poor facial nerve outcomes after 12 months (Fig. 1). For screening, a threshold MIB-1 index ≥ 4.5 was recommended and resulted in a sensitivity of 76.2% and a specificity of 73. 0% for predicting the likelihood of a poor (HB > 3) facial nerve outcome after 12 months. In total, 42.9% (12/28) of all patients with a MIB-1 index ≥ 5% had a good (HB ≤ 2) facial nerve outcome, whereas good facial nerve outcomes were achieved in 87.5% of the patients with a MIB-1 index < 5% (35/40). Pearson’s chi-squared test (two-sided) revealed these results were significantly different (*p* < 0.001).

The MIB-1 index was not correlated (Pearson’s correlation coefficient: *r* = − 0.066, *p* = 0.49) with tumour size, which was defined as the largest extrameatal tumour diameter on a post-contrast axial MRI.

CD68 and CD45 staining was performed in 66 and 35 cases, respectively. Positive staining of macrophages by CD68 was observed in 100% (31/31) of patients with a MIB-1 index ≥ 5% and 62.9% (22/35) of those with a MIB-1 index < 5% (Spearman’s rank correlation coefficient: *r* = 0.43, *p* = 0.021). Positive staining of lymphocytes by CD45 was observed in 100% of patients with a MIB-1 index ≥ 5% (14/14) and 66.6% of those with a MIB-1 index < 5% (14/21) (Spearman’s rank correlation coefficient: *r* = 0.63, *p* = 0.015).

### Multivariate analysis of facial nerve function at the 12-month follow-up

**Fig. 2:**
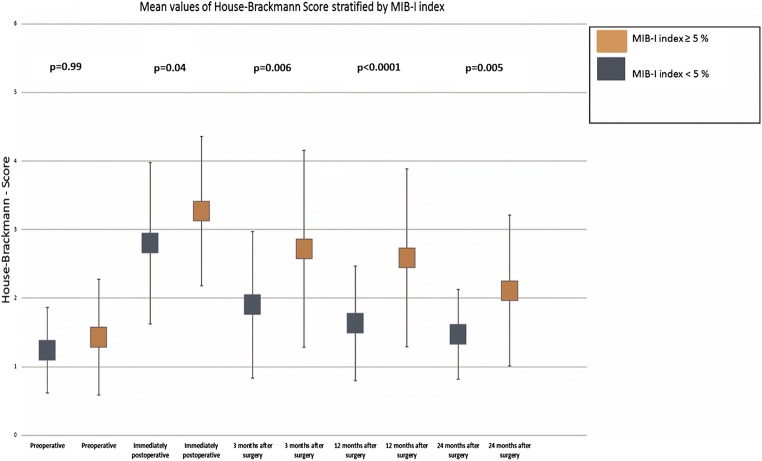
Scatter plots of House-Brackmann scale values stratified by MIB-1 index values “< 5%” and “ ≥ 5%”. The *Y*-axis represents the House-Brackmann scale. Scatter plots outline the mean and the standard deviation of House-Brackmann-scale values examined at baseline and postoperatively. Patients with an MIB-1 index < 5% are shown in the blue box, while those with an MIB-1 index ≥ 5% are shown in an orange box

Significantly more patients with a MIB-1 index ≥ 5% had poor HB scores throughout the whole postoperative follow-up period (Fisher’s exact test (two-sided), Table 3). A binary logistic regression analysis that included EoR, tumour size class, positioning, and the MIB-1 index revealed that a MIB-1 index ≥ 5% was a significant predictor of worse facial nerve function after 1 year (*p* < 0.001, odds ratio 14.0, 95% CI 3.20–61.14). The facial nerve outcomes achieved at the follow-up examinations in patients stratified by the MIB-1 index are shown in Fig. 2. Tumour size class 3 was also a significant factor (*p* = 0.01, odds ratio 14.56, 95% CI 1.87–113.43). The results of binary logistic regression analysis are summarised in Table 4.

**Table 3:** MIB-1 index and statistical facial nerve palsy analysis

	MIB-1 index ≥ 5%	MIB-1 index < 5%	*p* values
HB good (≤ 2) preoperatively	41/44 (93.2%)	66/70 (94.3%)	0.99
HB good (≤ 2) postoperatively	9/44 (20.5%)	28/70 (40.0%)	0.04
HB good (≤ 2) at 3 months	12/29 (41.4%)	31/41 (75.6%)	0.006
HB good (≤ 2) at 12 months	13/29 (44.8%)	35/40 (87.5%)	< 0.0001
HB good (≤ 2) at 24 months	11/19 (57.9%)	33/36 (91.7%)	0.005

**Table 4:** Binary logistic regression analysis of predictors of poor House-Brackmann scale outcomes at 12 months after surgery

Predictor	Wald	OR	95% CI	*p* value
Lateral positioning	0.92	1.96	0.49–7.81	0.34
Gross total resection	0.30	1.48	0.37–6.00	0.58
Tumour size class 3 (> 4 cm)	6.53	14.56	1.87–113.43	0.011
MIB-1 index ≥ 5%	12.30	14.0	3.20–61.14	< 0.001

## Discussion

This study investigated potential predictors of postoperative facial nerve function after vestibular schwannoma surgery. We found that an MIB-1 index of 5% or greater is an independent predictor of a poor postoperative facial nerve function after 12 months. The presence of CD68^+^ and CD45^+^ inflammatory cells is highly correlated with tumour proliferative activity in sporadic vestibular schwannomas.

### Extent of resection

In the last decade, philosophy regarding the treatment of VS has changed. Currently, an increasing number of centres prefer procedures aimed at the protection of the facial nerve to complete resection of the tumour [2, 6]. In our comparison of GTR versus STR, the facial nerve outcomes at 1 year after surgery were better when small tumour remnants were left behind to save the facial nerve. Consequently, the facial nerve seems to have the ability to recover within the first year. As previously proposed, postoperative stereotactic radiosurgery of tumour remnants should be initiated after 1 year [24]. The overall rate of recurrence in our 5-year follow-up group of 62 patients was 9.7%. This rate is consistent with previous studies that reported rates of recurrence of 8.8% or 10.71% for a subtotal resection [38, 39]. The rate of recurrence was not significantly different between GTR and STR in our Kaplan-Meier analysis. Nakatomi et al. [30] found in their analysis of vestibular schwannoma surgery that subtotal resection results in an 11-fold greater risk of recurrence compared with patients treated with gross total resection. Subtotal resection was defined in their study as anything less than a complete resection, which implies that also a very small extent of resection was included in this arm. Furthermore, Monfared et al. [28] analysed the role of extent of resection in 73 patients with large VSs, which were defined as tumours measuring 2.5 cm or larger in the cerebellopontine angle in this prospective study. They have found that the risk of tumour regrowth was 3 times higher after a subtotal resection compared with gross total resection and near total resection in the univariate analysis. Another retrospective study by Breshears et al. [8] investigated the role of the residual tumour volume after a subtotal resection in a cohort of 66 patients. They have shown that the volume of the residual tumour tissue was significantly associated with the recurrence of VSs. A tumour volume of 1.39 cm^3^ or higher was found to be associated with tumour regrowth. Additionally, the location of residual tumour tissue within the internal auditory canal was also significantly associated with tumour regrowth in their multivariate analysis. The extent of resection did not differ between their patients with and without recurrence in this study. Despite the large amounts of data suggesting gross total resection of vestibular schwannomas as the treatment of choice, Sughrue et al. [38] showed in a prospective series of 772 patients, who underwent either gross total, near total, or subtotal microsurgical resection of vestibular schwannomas, no significant relationship between the extent of resection and tumour recurrence. Limitation of this study is the long time period between 1984 and 2009 which might have led to some source of performance bias due to changes in operative techniques, availability of neuronavigation, different skills, and experience of the surgeons in this study. Furthermore, today’s follow-up imaging techniques using high-field (1.5 or 3.0 T) MRI were not available in this time period.

Consequently, direct comparison of rates of tumour recurrence is limited by heterogeneous definitions of the extent of resection in literature and the various postoperative therapy regimens as far as the adjuvant gamma-knife radiosurgery of residual tumour tissue is concerned.

In both resection groups in our study, recurrences occurred in the fifth year after VS surgery. These findings are in accordance with those of other studies that reported recurrence within the fifth postoperative year [6].

All in all, prospective randomised trials with stringent definitions of the extent of resection and the postoperative adjuvant treatment regimen are needed to elucidate the exact impact of the extent of resection and the residual tumour volume on the recurrence of vestibular schwannomas.

### Tumour sizes

Tumour size class 3 (> 4 cm), also called giant VS, was a significant predictor of worse HB outcomes after 1 year (compared with class 1, *p* = 0.049, and compared with class 2, *p =* 0.002). Multivariate analysis showed that it was also an independent factor that influenced facial nerve outcomes (*p* = 0.011). Only 40% of patients suffering from giant VS had a good HB after 2 years. Our data are in agreement with those presented in other reports about giant VS. Consequently, in the treatment of giant VS, facial nerve preservation should be prioritised over total tumour resection [37, 47]. Because of its higher incidence of postoperative poor HB scores, the rate of facial nerve palsy–induced morbidity was much higher in class 3 than in classes 1 and 2 (15.8% vs. 6.3% vs. 0%), and this should be discussed with affected patients.

### MIB-1 proliferation index

The MIB-1 index was not significantly correlated with regrowth or recurrence despite our finding that the mean value of the MIB-1 index was slightly higher in the cases with recurrence. In the literature, the MIB-1 index has been associated with recurrence [22, 46]. A retrospective study of 17 patients by Iannella et al. [22] claimed that a MIB-1 index > 2.5% is a predictor of tumour regrowth and that these patients should be carefully monitored by radiological follow-up. Strong evidence for a significant association between the MIB-1 index and tumour recurrence in vestibular schwannoma is provided in the study by Panigrahi et al. [32] which investigated 144 patients who underwent surgical excision for sporadic vestibular schwannoma. It was suggested that an MIB-1 index of 3.5% or greater was the only significant predictor of tumour recurrence. A limitation of this study is the short time of follow-up at a mean of 37.99 months. However, in our series of 118 patients, we could not find a significant association between tumour recurrence and the MIB-1 index. MIB-1 index and the potential role of recurrence are difficult to elucidate in the literature because of the various treatment protocols as far as radiosurgery after a subtotal resection is concerned. The patients who underwent a subtotal resection in our study were not routinely treated with radiosurgery for residual tumour remnants. Secondary malignant transformation of vestibular schwannomas with an elevated MIB-1 index who underwent subtotal resection and gamma-knife surgery is discussed in the literature. Yanamandala et al. [45], in 2013, described a case report of a 46-year-old woman with the initial diagnosis of a benign VS with an elevated MIB-1 index of 5.7%. The patient underwent a subtotal resection which was followed by gamma-knife radiosurgery for the residual tumour tissue. At 43 months follow-up examination, the patient had a recurrent VS and repeated subtotal resection was performed which revealed a VS with an increased MIB-1 index of 7.4%. After a third resection of this tumour because of recurrence, a frank transformation to malignant tumour with an MIB-1 index of 33.8% was observed. Consequently, a potential increased risk of recurrence and malignant transformation of VS with an initial elevated MIB-1 index secondary to adjuvant gamma-knife radiosurgery is discussed in the literature [43, 45].

In our study, we have found a significant correlation between MIB-1 index values and HB outcome. Although 42.9% of the patients with a MIB-1 index ≥ 5% had a good HB score after 1 year, the patients with a MIB-1 index < 5% achieved significantly superior findings in the uni- and multivariate analyses, with a good HB score obtained in 87.5% of 40 cases (*p* < 0.001). This correlation between the MIB-1 index and facial nerve outcomes has not been mentioned in previous studies with regard to the known confounders such as extent of resection, tumour size, and patient positioning. It has been reported that there is no significant correlation between the duration of general clinical symptoms of VS and MIB-1 index values [31]. In contrast to our findings, Charabi et al. [10, 11] evaluated cohorts of 21 and 124 patients with acoustic neuromas and found a significant inverse correlation between preoperative symptom duration and the MIB-1 index that was independent of tumour size in which patients with a short preoperative period of symptoms had a higher MIB-1 index.

Concerning pathophysiology, it can be assumed that local residual cells with higher mitotic activity driven by inflammation will disturb the local function of the facial nerve by contributing to the formation of scars. Lewis et al. [27] suggested that inflammation is a relevant parameter when evaluating the growth of vestibular schwannomas. Positive correlations between higher MIB-1 index values and the presence of CD68-positive macrophages and CD45-positive lymphocytes were also observed in our study. In an investigation by Lewis et al. [27], tumour tissues obtained from 8 sporadic VSs were immunohistochemically analysed, and the growing tumours were found to exhibit significantly higher proportions of Ki-67/Iba1 (ionised calcium-binding adapter molecule)-positive cells. Additionally, programmed cell death 1 ligand 1 (PD-L1) expression was recently described in VSs [5], and it was suggested that PD-L1 expression, tumour-associated macrophages, and rapid tumour growth could be correlated with each other. Paracrine cellular communication between tumour-associated macrophages and Schwann cells seems to explain the decreased anti-tumour effect of tumour-associated macrophages. Furthermore, COX2 expression was significantly associated with higher MIB-1 expression [4]. Scar formation is a known component of prolonged inflammatory reactions, which is also mediated by COX2 [42]. Therefore, a potential explanation for the poor facial nerve outcome of our patients with increased MIB-1 indices could be scar formation, which is mediated by inflammatory reactions. Surgical dissection of these tumours might be more challenging due to adhesions to the facial nerve.

### Limitations and future directions

The main limitation of this study is its retrospective nature, given the greater potential for missing data as far as follow-up examinations are concerned. There are some pitfalls associated with specimen sampling of CNS tumours with regard to the evaluation of the MIB-1 index. In partially or subtotally resected tumours, the sampled neoplastic tissue does not necessarily contain the highest proliferative activity in the area [13]. Furthermore, there was interobserver variability regarding the determination of the MIB-1 index in our collected data because of the long time period that was investigated. Additionally, a digital image analysis system was not used to determine the MIB-1 labelling index, although this might be a more objective approach because it allows a greater number of microscopic fields to be analysed [25].

All in all, the literature concerning inflammatory processes in sporadic VS and our data could contribute to therapeutic decision-making. Future studies are needed to investigate the potential association of proliferative activity with COX2 expression and potential postoperative therapeutic implications which inhibit this pathway. In animal models, it was observed that the use of acetylsalicylic acid is beneficial to attenuate demyelination and increase the diameter of myelin sheaths in regenerating axons after a peripheral nerve injury with inflammatory reactions [15]. Furthermore, rapid immunohistochemical methods based on alternating current electric fields are available to facilitate the interaction between antigen and antibody. These methods make it possible to determine the MIB-1 indices and the tumour proliferation activity intraoperatively [29, 41]. Therefore, new treatment modalities that can downregulate inflammatory processes and enable the construction of highly personalised therapies, which can be used immediately after VS surgery in high-risk patients with worse postoperative facial nerve outcomes, are needed.

## Conclusion

In addition to previously identified predictors, such as tumour size, the extent of resection and surgical positioning, of long-term facial nerve function after VS surgery, an MIB-1 index ≥ 5% seems to be an independent predictor of persistent facial nerve palsy.
